# Evidence of metachronous development of ovarian teratomas: a case report of bilateral mature cystic teratomas of the ovaries and systematic literature review

**DOI:** 10.1186/s13048-017-0313-8

**Published:** 2017-03-14

**Authors:** Wen-Chung Wang, Yen-Chein Lai

**Affiliations:** 1 Department of Obstetrics and Gynecology, Jen-Ai Hospital, Taichung, Taiwan; 2 Department of Medical Laboratory and Biotechnology, Chung Shan Medical University, No.110, Sec. 1, Chien Kuo N. Road, Taichung, 402 Taiwan, Republic of China

**Keywords:** Mature cystic teratoma, Metachronous, Short tandem repeat analysis, Multiplex ligation-dependent probe amplification analysis, Meiosis, Parthenogenesis

## Abstract

**Background:**

Mature cystic teratomas are usually found in the ovaries. They are bilateral in 10 to 15% of cases and multiple cystic teratomas may be present in one ovary. The aim of this study is to clarify if development of mature cystic teratomas of the ovaries in a single host is metachronous or due to autoimplant or recurrence.

**Case presentation:**

We report a woman with bilateral mature cystic teratomas of the ovaries. DNA profiles of these teratomas were investigated via short tandem repeat (STR) analysis and methylation statuses were determined via methylation sensitive multiplex ligation-dependent probe amplification methods. The results showed that the cystic teratomas originated from different stages of oogonia or primary oocyte before germinal vesicle stage failure of meiosis I in female gametogenesis.

Potentially relevant literature was searched in PubMed database. Cases of bilateral or multiple mature cystic teratomas of the ovaries were analyzed. To date, there has been no reported case of multiple mature cystic teratomas in which clarification of the origin was achieved using molecular genetic methods.

**Conclusions:**

The results of this case study provide evidence of metachronous development of mature cystic teratomas of the ovaries and may serve as a reference in the management of patients following laparoscopic cystectomy.

**Electronic supplementary material:**

The online version of this article (doi:10.1186/s13048-017-0313-8) contains supplementary material, which is available to authorized users.

## Background

Most germ cell tumors occur in a variety of sites, both gonadal and extragonadal [1]. Teratoma is a benign germ cell tumor of more than one cell type, originating from more than one germ layer [2]. One of the most common locations is the ovary [3]. Benign cystic teratomas (dermoid cysts) of the ovary are composed of mature histologic structures of ectodermal, mesodermal and endodermal origin [4]. They make up 10 to 15% of all ovarian tumors and tend to occur at relatively early age [5]. They are bilateral in 10 to 15% of cases and multiple cystic teratomas may be present in one ovary [6, 7]. A recent study has reported that more than one fifth of children with ovarian mature teratoma develop metachronous benign tumor in the contralateral ovary [8]. There have been two additional reports of metachronous mature cystic teratomas [9, 10]. This raises the question of whether these tumors are the result of autoimplant, or are recurrent synchronous or metachronous. There is currently no suitable method of differentiation available. In the 1980s, centromere marks were used to differentiate the origins of bilateral teratomas in a single patient [11]. Previous studies have suggested that multiple ovarian dermoid cysts in a single host originate from different germ cells via diverse mechanisms based on older molecular methods such as slab gels and isotopic labeling [11, 12]. Further studies are needed to clarify the origin of benign mature cystic teratomas of the ovary using more “modern” methodology such as automated capillary sequencing machines in which radioisotopic tags are displaced by fluorescent labels.

Our previous study demonstrated that the origin of mature cystic teratomas of the ovary is oogonia or primary oocyte before germinal vesicle stage failure of meiosis I [13]. The present case study, conducted in Taiwan, is of a 34-year-old woman with bilateral mature cystic teratomas of the ovaries. We investigated the DNA profiles of the teratomas using short tandem repeat (STR) analysis and methylation statuses using methylation sensitive multiplex ligation-dependent probe amplification methods. The aim of this study is to clarify if development of mature cystic teratomas of the ovaries in a single host is metachronous or due to autoimplant or recurrence.

## Case presentation

A woman presenting with bilateral mature cystic teratomas of the ovaries came to our attention during our previous study [13]. Tumors were considered to be mature without immature components after examination of multiple sections. There was no evidence of malignancy. The pathological diagnosis was benign mature cystic teratoma. The Institutional Review Board of Jen-Ai Hospital approved all procedures and informed consent was obtained prior to collecting her genetic material for the study. DNA was extracted from paraffin-embedded sections of the left and right teratoma tissues with QIAamp DNA FFPE Tissue Kit (Qiagen), according to the manufacturer’s instructions. DNA from each teratoma was taken from solid nodule within inner site of tumor.

**Fig. 1:**
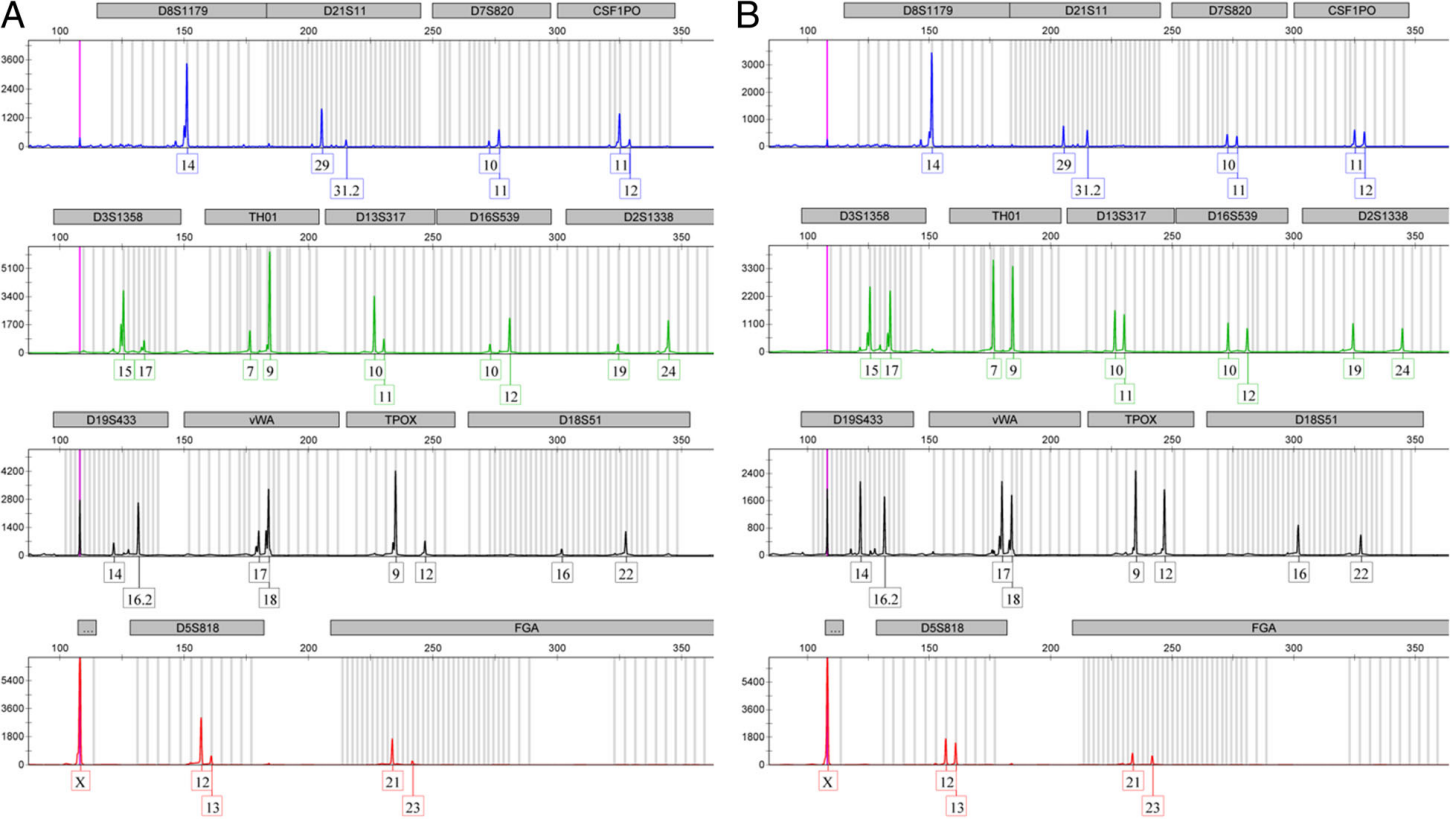
Electropherogram of the DNA profiles of right (**a**) and left (**b**) mature cystic teratomas of the ovaries

**Fig. 2:**
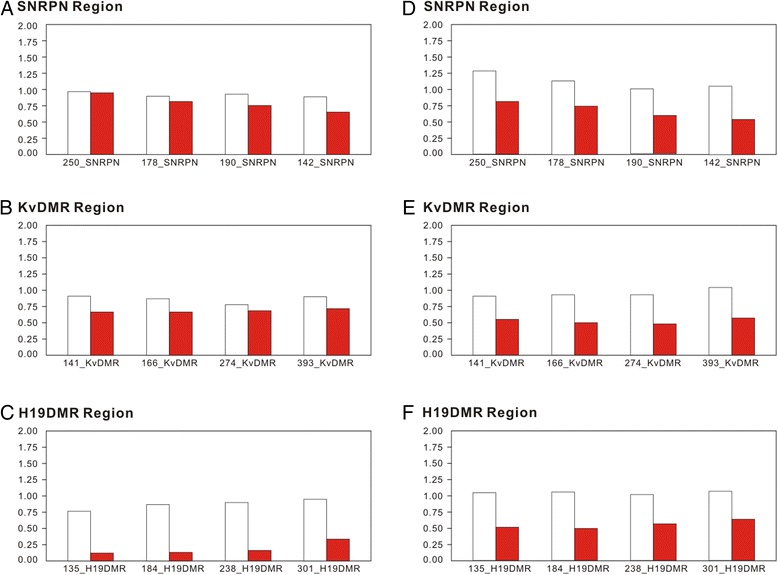
The copy numbers and methylation ratios of right (**a**, **b**, and **c**) and left (**d**, **e**, and **f**) mature cystic teratomas of the ovaries. Open column represents the copy number ratio and solid red column represents the methylation ratio. Panels are as follows: (**a**, **d**) four 15q11 *SNRPN* probes; (**b**, **e**) four 11p15 KvDMR probes; (**c**, **f**) four 11p15 H19DMR probes

We determined DNA profiles on STR analysis, using AmpFLSTR® SGM Plus, Profiler PCR amplification kits according to the methods described by Wang et al. [14]. As shown in Fig. 1a, the DNA profiles of right tumor tissue revealed heterozygosity in 14 of the 15 analyzed STRs. DNA profiles of left tumor tissue were similar, as shown in Fig. 1b. The right tumor showed more significant loss of heterozygosity in 13 of the 14 analyzed STRs than the left tumor (Table 1). Moreover, the copy number ratios and methylation statuses of the *Hha*I sites in the *SNRPN*, KvDMR and H19DMR probes of these mature cystic teratomas of the ovaries are presented in Fig. 2 and Additional file 1: Table S1. They were obtained from methylation sensitive multiplex ligation-dependent probe amplification analysis according to previously described methods [13]. The average copy number ratios and methylation statuses of the *SNRPN*, KvDMR and H19DMR probes are presented in Table 2. *SNRPN* and *KCNQ1OT1* are maternally imprinted genes, with the maternal allele methylated in somatic cells. There were varying degrees of hypermethylation of *SNRPN* and KvDMR in the presence of maternal uniparental disomy in both mature cystic teratomas (Table 2). This suggests that methylation imprint of *SNRPN* and KvDMR is well established and, as expected, is from maternal chromosomes at various stages of female gametogenesis. *H19* is a paternally imprinted gene, with the paternal allele methylated in somatic cells. H19DMR showed significant hypomethylation in the right mature cystic teratoma (Table 2).

**Table 1:** Loss of heterozygosity in 15 STR loci of mature cystic teratomas of the ovary

STR Loci	Location	Alleles	Right R^a^	Left R^a^
TPOX	2p23-2per	9, 12	5.44^c^	1.29^b^
D2S1338	2q35-37.1	19, 24	2.02^c^	1.18
D3S1358	3p21.31	15, 17	0.27^c^	1.08
FGA	4q28	21, 23	1.25^b^	1.21
D5S818	5q21–31	12, 13	5.05^c^	1.18
CSF1PO	5q33.3–34	11, 12	4.55^c^	1.04
D7S820	7q11.21–22	10, 11	0.33^c^	1.16
D8S1179	8q24.1–24.2	14, 14	ND	ND
TH01	11p15.5	7, 9	0.22^c^	1.06
vWA	12p12-pter	17, 18	0.36^c^	1.18
D13S317	13q22–31	10, 11	4.04^c^	1.10
D16S539	16q24-qter	10, 12	0.25^c^	1.18
D18S51	18q21.3	16, 22	0.30^c^	1.45^b^
D19S433	19q12–13.1	14, 16.2	0.26^c^	1.24
D21S11	21q11.2-q21	29, 31.2	5.45^c^	1.26^b^

^a^R = area T1/ area T2; ^b^Loss of heterozygosity is positive when R ≥ 1.25 or ≤ 0.8 (ie., 20% change); ^c^Loss of heterozygosity is more significant when R ≥ 2.0 or ≤ 0.5 (ie., 50% change)

**Table 2:** Average copy numbers and methylation ratios of the 15q11 *SNRPN,* 11p15 KvDMR and H19DMR probes in mature cystic teratomas of the ovaries

Gene loci	Right	Left
*SNRPN* Region		
Copy No. Ratio	0.925 ± 0.038	1.125 ± 0.125
Met. Ratio	0.796 ± 0.123	0.675 ± 0.130
% Met.	85.89 ± 10.46	59.71 ± 6.46
KvDMR Region		
Copy No. Ratio	0.867 ± 0.055	0.956 ± 0.058
Met. Ratio	0.688 ± 0.024	0.529 ± 0.044
% Met.	79.51 ± 5.88	55.33 ± 3.72
H19DMR Region		
Copy No. Ratio	0.884 ± 0.062	1.050 ± 0.023
Met. Ratio	0.188 ± 0.098	0.554 ± 0.065
% Met.	20.90 ± 9.44	52.80 ± 5.93

Data are presented as mean ± SD

## Literature review

Published articles were systematically searched in the PubMed database with human-source restriction using the search terms: “ovary” and “bilateral or multiple or recurrent mature cystic teratoma”. The inclusion criteria were papers written in English or Chinese concerning the origin of mature cystic teratomas of the ovary. The exclusion criteria were articles written in a language other than English or Chinese and cases of mature cystic teratomas of the ovary without extractable data.

Finally, 51 articles describing 3194 cases of mature cystic teratomas of the ovary were included (Additional file 2: Table S2). Based on case reports, most (32/39) were synchronous bilateral and 4/39 were metachronous bilateral [9, 15–17], including 2 cases of ovarian teratoma with extraovarian teratoma [9, 17]. Only 5/39 of the reported cases were due to recurrence [15, 17–19], including 1 case of ovarian teratoma with extraovarian teratoma [17]. In terms of serial cases, in 16 articles were described 3155 cases of mature cystic teratomas of the ovary. Among them, 236 were synchronous bilateral [20–31], 55 were metachronous bilateral [6, 32, 33], and 29 were due to recurrence [6, 32–34]. To date, there has been no published case in which clarification of the origin was achieved with molecular genetic methods.

## Discussion

The most accepted theory regarding mature cystic teratomas of the ovary is that they are of parthenogenetic origin from oocyte after the completion of first division [35]. In this study, the heterozygosity in our DNA profiling data did not support a parthenogenetic origin of mature cystic teratomas of the ovary (Table 2 and Fig. 1), which is consistent with the findings of our previous study [13]. The heterozygosity in our DNA profiling data was also consistent with that demonstrated by Nomura et al. [12]. In their study, multiple ovarian dermoid cysts in a single host originated from different germ cells via diverse mechanisms [12]. However, our findings were inconsistent with those of Carritt et al., which revealed homozygosity among eight enzyme and centromere markers in three teratomas using older molecular methods [11]. Based on “modern” methodology, small minor alleles in heterozygous loci with significant loss of heterozygosity were found in D21S11, D7S820, CSF1PO, D3S1358, D16S539, D2S1338, D18S51, D5S818, and FGA in the right tumor (Table 1 and Fig. 1). With older molecular methods, such as polyacrylamide slab gel electrophoresis, it is possible to miss these small minor alleles and to misclassify them as homozygous using DNA polymorphisms and enzyme markers [11, 36, 37].

**Fig. 3:**
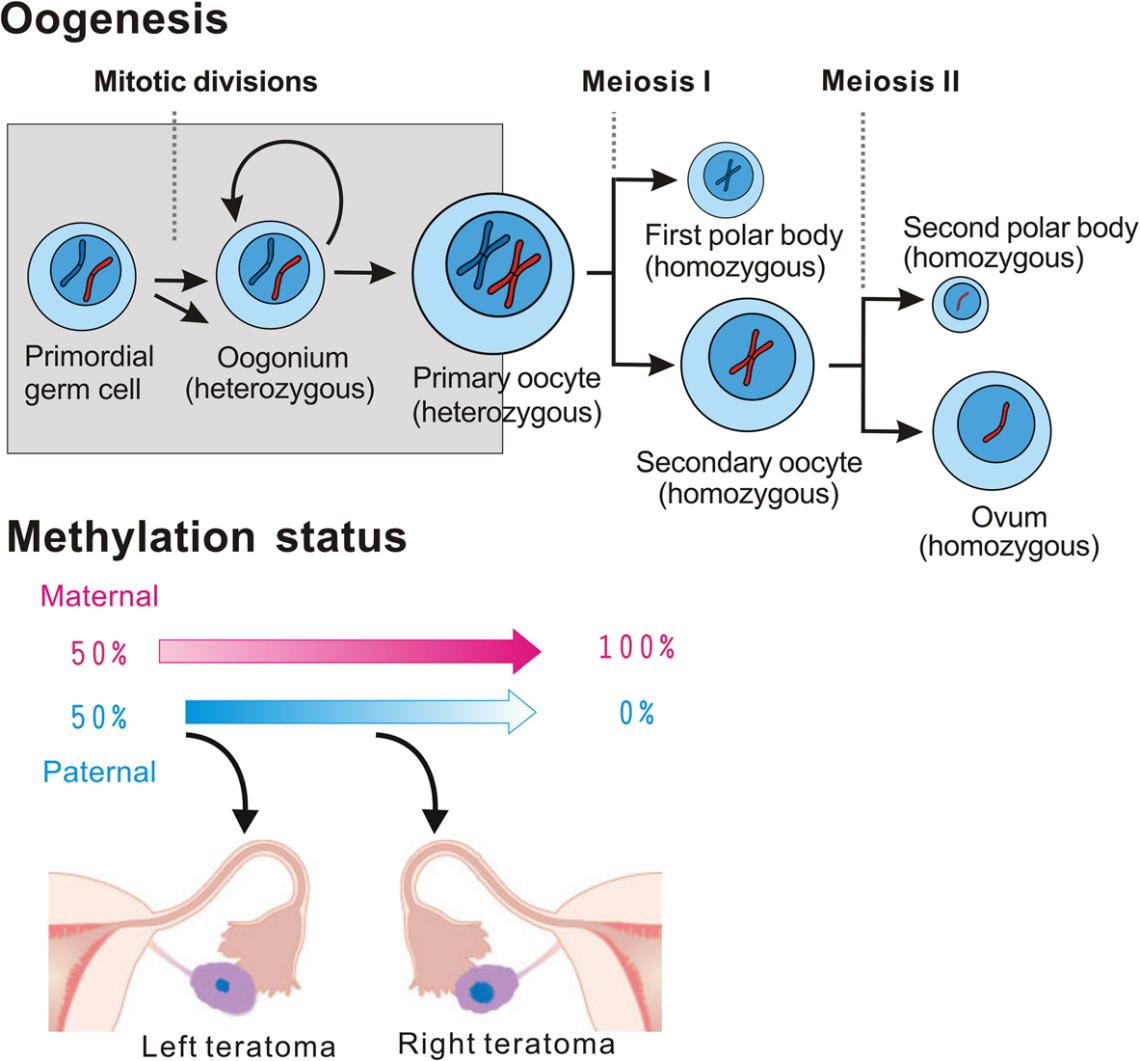
Methylation statuses of the bilateral mature cystic teratomas of the ovaries during oogenesis. As an ovum develops in the ovary its paternal methylation is erased. The primary oocyte presents at birth and the secondary oocyte is the stage released by the ovary during ovulation. Primary oocyte production begins before birth and is indicated by shading

During oogenesis, previous somatic epigenetic modifications are erased and new sex-specific epigenetic markers are acquired [38]. In our previous study, we suggested that the varying degrees of hypomethylation of H19DMR and hypermethylation of *SNRPN* and KvDMR are dependent on the stage of oogenesis from which each tumor arises [13]. In this study, we postulated that the origins of left cystic teratoma are in early oogenesis in paternal methylation, which has not begun or has just begun to be erased with establishment of maternal methylation (Fig. 3). The right cystic teratoma was more than midway through the genomic imprinting progress.

There are four possible mechanisms for development of bilateral mature cystic teratomas of the ovaries. The first is metachronous development in which the original tumor cells are arrested at different stages of oogenesis and development is independently restarted due to unknown stimulant. The second is synchronous development in which the same original tumor cells are arrested at same stage of oogenesis and development is simultaneously restarted due to unknown stimulant. The third is that one of the mature cystic teratomas is primary tumor and one is the result of metastasis from primary tumor. The fourth is recurrence due to residual teratoma or spreading during operation. There is no molecular evidence to clearly support any one of these mechanisms. If due to the first mechanism, bilateral mature cystic teratomas of the ovaries should present in different stages in the imprinted gene process. If due to any of the remaining mechanisms, bilateral mature cystic teratomas should present in the same stage of imprinted gene process. In this study, bilateral mature cystic teratomas presented in different stages of imprinted gene process, which supports the first explanation. Maternal methylation of *SNRPN* and KvDMR progressively increased and paternal methylation of H19DMR progressively decreased, as the stage of germ cells from which the tumors were presumed to have arisen increased.

The management of ovarian mature cystic teratomas has shifted from oophorectomy to laparoscopic cystectomy [39]. However, there is high frequency of spillage of content during laparoscopic cystectomy [40]. Chemical peritonitis is the complication of concern and can be prevented by copious lavage [41]. Late recurrent and parasitic teratomas have recently been reported [19, 42]. However, the question remains whether such tumors are really recurrent or the result of metachronous development. Our methods can be applied in attempting to answer that question. Further studies should include analyses of recurrent teratomas using the present molecular genetic methods. This would allow for differentiation of recurrent tumor due to residual teratoma from previous surgery, which has spread from the surgical site, and newly developed teratoma.

## Conclusions

The present case study, conducted in Taiwan, is of a woman with bilateral mature cystic teratomas of the ovaries. DNA profiling and methylation sensitive multiplex ligation-dependent probe amplification methods revealed that these mature cystic teratomas originated from different stages of oogonia or primary oocyte before germinal vesicle stage failure of meiosis I in female gametogenesis. The results of this case study provide evidence of metachronous development of mature cystic teratomas of the ovaries and may serve as a reference in the management of patients following laparoscopic cystectomy.

## Additional file

Additional file 1: Table S1. Raw data on copy numbers and methylation ratios of the 15q11 *SNRPN,* 11p15 KvDMR and H19DMR probes in mature cystic teratomas of the ovaries. (DOCX 14 kb)
Additional file 2: Table S2. 51 articles describing 3194 cases of bilateral or multiple mature cystic teratomas of the ovary were stratified into recurrent, synchronous and metachronous development. (DOCX 67 kb)

## Abbreviations

DNA: Deoxyribonucleic acid
PCR: Polymerase chain reaction
*SNRPN*: Small nuclear ribonucleoprotein polypeptide N
STR: Short tandem repeat

## References

[1] Isaacs H (2004). Perinatal (fetal and neonatal) germ cell tumors. J Pediatr Surg.

[2] Oliveira FG, Dozortsev D, Diamond MP, Fracasso A, Abdelmassih S, Abdelmassih V (2004). Evidence of parthenogenetic origin of ovarian teratoma: case report. Hum Reprod.

[3] Crum CP, Cohan RS, Kumar V, Collins T (1999). Female genital tract--ovarian tumors. Robbins pathologic basis of disease.

[4] Peterson CM, Buckley C, Holley S, Menias CO (2012). Teratomas: a multimodality review. Curr Probl Diagn Radiol.

[5] Bollen N, Camus M, Tournaye H, De Munck L, Devroey P (1992). Laparoscopic removal of benign mature teratoma. Hum Reprod.

[6] Doss N, Forney JP, Vellios F, Nalick RH (1977). Covert bilaterality of mature ovarian teratomas. Obstet Gynecol.

[7] Pepe F, Lo Monaco S, Rapisarda F, Raciti G, Genovese C, Pepe P (2014). An unusual case of multiple and bilateral ovarian dermoid cysts. Case report. G Chir.

[8] Taskinen S, Urtane A, Fagerholm R, Lohi J, Taskinen M (2014). Metachronous benign ovarian tumors are not uncommon in children. J Pediatr Surg.

[9] Okino H, Koga Y, Tsuneyoshi M, Takeda S (2006). Metachronous mature cystic teratomas in the left ovary and bilateral diaphragm: report of a case. Surg Today.

[10] Gobbi D, Fascetti Leon F, Aquino A, Melchionda F, Lima M (2013). Metachronous bilateral ovarian teratoma: a germ-line familial disorder and review of surgical management options. J Pediatr Adolesc Gynecol.

[11] Carritt B, Parrington JM, Welch HM, Povey S (1982). Diverse origins of multiple ovarian teratomas in a single individual. Proc Natl Acad Sci U S A.

[12] Nomura K, Ohama K, Okamoto E, Fujiwara A (1983). Cytogenetic studies of multiple ovarian dermoid cysts in a single host. Nihon Sanka Fujinka Gakkai Zasshi.

[13] Wang WC, Lai YC (2016). Genetic analysis results of mature cystic teratomas of the ovary in Taiwan disagree with the previous origin theory of this tumor. Hum Pathol.

[14] Wang WC, Lee MS, Ko JL, Lai YC (2011). Origin of uterine teratoma differs from that of ovarian teratoma: a case of uterine mature cystic teratoma. Int J Gynecol Pathol.

[15] Chang CF, Lin CK (2014). A case of recurrent, bilateral ovarian mature teratoma in a young woman. BMC Womens Health.

[16] Sinha R, Sethi S, Mahajan C, Bindra V (2010). Multiple and bilateral dermoids: a case report. J Minim Invasive Gynecol.

[17] Kommoss F, Emond J, Hast J, Talerman A (1990). Ruptured mature cystic teratoma of the ovary with recurrence in the liver and colon 17 years later. A case report. J Reprod Med.

[18] Engel T, Greeley AV, Sweeney WJ (1965). Recurrent dermoid cysts of the ovary. Report of 2 cases. Obstet Gynecol.

[19] Sinha R, Sundaram M, Lakhotia S (2009). Multiple intraabdominal parasitic cystic teratomas. J Minim Invasive Gynecol.

[20] Coskun A, Kiran G, Ozdemir O (2008). CA 19–9 can be a useful tumor marker in ovarian dermoid cysts. Clin Exp Obstet Gynecol.

[21] Vang R, Gown AM, Zhao C, Barry TS, Isacson C, Richardson MS (2007). Ovarian mucinous tumors associated with mature cystic teratomas: morphologic and immunohistochemical analysis identifies a subset of potential teratomatous origin that shares features of lower gastrointestinal tract mucinous tumors more commonly encountered as secondary tumors in the ovary. Am J Surg Pathol.

[22] Dede M, Gungor S, Yenen MC, Alanbay I, Duru NK, Hasimi A (2006). CA19-9 may have clinical significance in mature cystic teratomas of the ovary. Int J Gynecol Cancer.

[23] Papadias K, Kairi-Vassilatou E, Kontogiani-Katsaros K, Argeitis J, Kondis-Pafitis A, Greatsas G (2005). Teratomas of the ovary: a clinico-pathological evaluation of 87 patients from one institution during a 10-year period. Eur J Gynaecol Oncol.

[24] Koçak M, Dilbaz B, Ozturk N, Dede S, Altay M, Dilbaz S (2004). Laparoscopic management of ovarian dermoid cysts: a review of 47 cases. Ann Saudi Med.

[25] Sah SP, Uprety D, Rani S (2004). Germ cell tumors of the ovary: a clinicopathologic study of 121 cases from Nepal. J Obstet Gynaecol Res.

[26] Al-Fozan H, Glassman J, Caspi B, Appelman Z, Tulandi T (2003). Lateral distribution of ovarian dermoid cyst. J Am Assoc Gynecol Laparosc.

[27] Wu RT, Torng PL, Chang DY, Chen CK, Chen RJ, Lin MC (1996). Mature cystic teratoma of the ovary: a clinicopathologic study of 283 cases. Zhonghua Yi Xue Za Zhi (Taipei).

[28] Ayhan A, Aksu T, Develioglu O, Tuncer ZS, Ayhan A (1991). Complications and bilaterality of mature ovarian teratomas (clinicopathological evaluation of 286 cases). Aust N Z J Obstet Gynaecol.

[29] Tarcoveanu E, Vasilescu A, Georgescu S, Danila N, Bradea C, Lupascu C (2012). Laparoscopic approach to ovarian dermoid cysts. Chirurgia (Bucur).

[30] Saks M, Deckardt R (1994). Laparoscopic Treatment of Benign Ovarian Dermoid Cysts. J Am Assoc Gynecol Laparosc.

[31] Malkasian GD, Dockerty MB, Symmonds RE (1967). Benign cystic teratomas. Obstet Gynecol.

[32] Anteby EY, Ron M, Revel A, Shimonovitz S, Ariel I, Hurwitz A (1994). Germ cell tumors of the ovary arising after dermoid cyst resection: a long-term follow-up study. Obstet Gynecol.

[33] Pepe F, Panella M, Pepe G, Panella P, Pennisi F, Arikian S (1986). Dermoid cysts of the ovary. Eur J Gynaecol Oncol.

[34] Song YN, Zhu L, Lang JH (2007). Recurrent mature ovarian teratomas: retrospective analysis of 20 cases. Zhonghua Yi Xue Za Zhi.

[35] Linder D, McCaw BK, Hecht F (1975). Parthenogenic origin of benign ovarian teratomas. N Engl J Med.

[36] Parrington JM, West LF, Povey S (1984). The origin of ovarian teratomas. J Med Genet.

[37] Deka R, Chakravarti A, Surti U, Hauselman E, Reefer J, Majumder PP (1990). Genetics and biology of human ovarian teratomas. II. Molecular analysis of origin of nondisjunction and gene-centromere mapping of chromosome I markers. Am J Hum Genet.

[38] Denomme MM, Mann MR (2012). Genomic imprints as a model for the analysis of epigenetic stability during assisted reproductive technologies. Reproduction.

[39] Sinha A, Ewies AA (2016). Ovarian mature cystic teratoma: Challenges of surgical management. Obstet Gynecol Int.

[40] Berg C, Berndorff U, Diedrich K, Malik E (2002). Laparoscopic management of ovarian dermoid cysts. A series of 83 cases. Arch Gynecol Obstet.

[41] Godinjak Z, Bilalovic N, Idrizbegovic E (2011). Laparoscopic treatment of ovarian dermoid cysts is a safe procedure. Bosn J Basic Med Sci.

[42] Harada M, Osuga Y, Fujimoto A, Fujimoto A, Fujii T, Yano T (2013). Predictive factors for recurrence of ovarian mature cystic teratomas after surgical excision. Eur J Obstet Gynecol Reprod Biol.

