# Supplementary material for: Evidence of metachronous development of ovarian teratomas: a case report of bilateral mature cystic teratomas of the ovaries and systematic literature review
Source: J Ovarian Res. 2017 Mar 14;10:17. doi: 10.1186/s13048-017-0313-8 (PMC5348818; doi:10.1186/s13048-017-0313-8)
Supplement: Additional file 1: Table S1. — Raw data on copy numbers and methylation ratios of the 15q11 SNRPN, 11p15 KvDMR and H19DMR probes in mature cystic teratomas of the ovaries. (DOCX 14 kb) [file 13048_2017_313_MOESM1_ESM.docx]

**Table S1** Raw data on copy numbers and methylation ratios of the 15q11 *SNRPN,* 11p15 KvDMR and H19DMR probes in mature cystic teratomas of the ovaries

|  | **Right** | |  | **Left** | |
| --- | --- | --- | --- | --- | --- |
| **Gene Loci** | Copy No. Ratio | Met. Ratio |  | % Methylation | Met. Ratio |
| ***SNRPN* Region** |  |  |  |  |  |
| 250_SNRPN CpG | 0.977 | 0.951 |  | 1.290 | 0.818 |
| 178_SNRPN CpG | 0.902 | 0.819 |  | 1.141 | 0.749 |
| 190_SNRPN CpG | 0.927 | 0.760 |  | 1.003 | 0.592 |
| 142_SNRPN CpG | 0.893 | 0.655 |  | 1.064 | 0.542 |
| **KvDMR Region** |  |  |  |  |  |
| 141_KvDMR | 0.900 | 0.673 |  | 0.917 | 0.555 |
| 166_KvDMR | 0.882 | 0.668 |  | 0.936 | 0.499 |
| 274_KvDMR | 0.786 | 0.689 |  | 0.929 | 0.485 |
| 393_KvDMR | 0.900 | 0.721 |  | 1.043 | 0.576 |
| **H19DMR Region** |  |  |  |  |  |
| 135_H19DMR | 0.807 | 0.125 |  | 1.043 | 0.515 |
| 184_H19DMR | 0.870 | 0.129 |  | 1.063 | 0.496 |
| 238_H19DMR | 0.905 | 0.166 |  | 1.021 | 0.565 |
| 301_H19DMR | 0.954 | 0.333 |  | 1.072 | 0.641 |
