# Supplementary material for: Evidence of metachronous development of ovarian teratomas: a case report of bilateral mature cystic teratomas of the ovaries and systematic literature review
Source: J Ovarian Res. 2017 Mar 14;10:17. doi: 10.1186/s13048-017-0313-8 (PMC5348818; doi:10.1186/s13048-017-0313-8)
Supplement: Additional file 2: Table S2. — 51 articles describing 3194 cases of bilateral or multiple mature cystic teratomas of the ovary were stratified into recurrent, synchronous and metachronous development. (DOCX 67 kb) [file 13048_2017_313_MOESM2_ESM.docx]

**Table S2** 51 articles describing 3194 cases of bilateral or multiple mature cystic teratomas of the ovary were stratified into recurrent, synchronous and metachronous development.

|  | No. | Synchronous | Metachronous | Recurrent |
| --- | --- | --- | --- | --- |
| Bilateral one case report | 25 | 1/1 [1-22] | 1/1 [23, 24] | 1/1 [23, 25] |
| Bilateral two case reports | 8 | 2/2 [26-28] |  | 2/2 [29] |
| Ovarian and extraovarian teratomas | 6 | 1/1 [30-33] | 1/1 [34, 35] | 1/1 [35] |
| Reports on serial cases | 3155 | 3/43 [36], 6/44 [37], 22/80 [38], 10/87 [39], 3/47 [40], 28/113 [41], 45/336 [42], 23/283 [43], 31/226 [44], 8/38 [45], 4/45 [46], 53/612 [47] | 18/99 [48], 10/105 [49], 27/213 [50] | 3/99 [48], 5/105 [49], 1/213 [50], 20/784 [51] |

**Supplemental References**

1. Chitrakar NS, Suwal S, Neupane S. Bilateral Ovarian Teratoma: One Parasitic Twisted In-situ and Another Parasitic at the Hepato Renal Space. J Nepal Health Res Counc. 2015;13:166-8.

2. Qiao PF, Gao Y, Niu GM. Struma ovarii accompanied by mature cystic teratoma of the other ovary: A case report and literature review. Oncol Lett. 2015;9:2053-2055.

3. Hakim MM, Abraham SM. Bilateral dermoid ovarian cyst in an adolescent girl. BMJ Case Rep. 2014;2014.

4. Pepe F, Lo Monaco S, Rapisarda F, Raciti G, Genovese C, Pepe P. An unusual case of multiple and bilateral ovarian dermoid cysts. Case report. G Chir. 2014;35:75-7.

5. Bolla D, Deseo N, Sturm A, Schoning A, Leimgruber C. Minilaparotomy a good option in specific cases: a case report of bilateral ovarian germ cell tumor. Case Rep Obstet Gynecol. 2012;2012:589568.

6. Menon S, Deodhar K, Rekhi B, John A, Maheshwari A. Unilateral malignant struma ovarii in a case of bilateral ovarian teratoma with raised CA-125 level: a rare case with treatment dilemmas. Indian J Pathol Microbiol. 2011;54:578-80.

7. Walid MS, Boddy MG. Bilateral dermoid cysts of the ovary in a pregnant woman: case report and review of the literature. Arch Gynecol Obstet. 2009;279:105-8.

8. Jona JZ, Burchby K, Vitamvas G. Castration-sparing management of an adolescent with huge bilateral cystic teratomas of the ovaries. J Pediatr Surg. 1988;23:973-4.

9. Sorensen SS, Balslev E. Uterus didelphys associated with bilateral dermoid cysts and polycystic ovarian disease. Acta Obstet Gynecol Scand. 1987;66:171-3.

10. Futterweit W, Scher J, Nunez AE, Strauss L, Rayfield EJ. A case of bilateral dermoid cysts, insulin resistance, and polycystic ovarian disease: association of ovarian tumors with polycystic ovaries with review of the literature. Mt Sinai J Med. 1983;50:251-5.

11. Gabbay-Moore M, Ovadia Y, Neri A. Accessory ovaries with bilateral dermoid cysts. Eur J Obstet Gynecol Reprod Biol. 1982;14:171-3.

12. Imperato-McGinley J, Peterson RE, Sturla E, Dawood Y, Bar RS. Primary amenorrhea associated with hirsutism, acanthosis nigricans, dermoid cysts of the ovaries and a new type of insulin resistance. Am J Med. 1978;65:389-95.

13. Aiman J, Nalick RH, Jacobs A, Porter JC, Edman CD, Vellios F, et al. The origin of androgen and estrogen in a virilized postmenopausal woman with bilateral benign cystic teratomas. Obstet Gynecol. 1977;49:695-704.

14. Mehra U, O'Connor T, Ostapowicz F, Cavanagh D. Pregnancy with bilateral ruptured benign cystic teratomas. Am J Obstet Gynecol. 1976;124:361-6.

15. Scharfman E. Bilateral dermoid cysts complicating pregnancy. West J Surg Obstet Gynecol. 1960;68:304-5.

16. Taylor CP. Benign cystic teratoma. Case report of pregnancy subsequent to bilateral ovarian resection of "dermoid cysts*.* Obstet Gynecol. 1959;14:523-6.

17. Bell HG. Pregnancy following bilateral cystectomy for large dermoids; report of a case. Obstet Gynecol. 1959;13:701-4.

18. Hofmeister FJ, Miller JP. Bilateral dermoid cysts complicating pregnancy; management; report of a case. Obstet Gynecol. 1956;7:684-8.

19. Ahnquist G. Pregnancy following bilateral dermoid cystectomy. Am J Obstet Gynecol. 1953;66:415-20.

20. Metcalfe EM. Bilateral ovarian dermoids in a girl of twelve. J Obstet Gynaecol Br Emp. 1947;54:657.

21. Kearney MS. Synchronous benign teratomas of the greater omentum and ovary. Case report. Br J Obstet Gynaecol. 1983;90:676-9.

22. Bournas N, Varras M, Kassanos D, Chrelias Ch, Tzaida O, Salamalekis E. Multiple dermoid cysts within the same ovary: our experience of a rare case with review of the literature. Clin Exp Obstet Gynecol. 2004;31:305-8.

23. Chang CF, Lin CK. A case of recurrent, bilateral ovarian mature teratoma in a young woman. BMC Womens Health. 2014;14:57.

24. Sinha R, Sethi S, Mahajan C, Bindra V. Multiple and bilateral dermoids: a case report. J Minim Invasive Gynecol. 2010;17:235-8.

25. Sinha R, Sundaram M, Lakhotia S. Multiple intraabdominal parasitic cystic teratomas. J Minim Invasive Gynecol. 2009;16:789-91.

26. Takeda A, Imoto S, Nakamura H. Management of pediatric and adolescent adnexal masses by gasless laparoendoscopic single-site surgery. Eur J Obstet Gynecol Reprod Biol. 2014;181:66-71.

27. Ozcan R, Kuruoglu S, Dervisoglu S, Elicevik M, Emir H, Buyukunal C. Ovary-sparing surgery for teratomas in children. Pediatr Surg Int. 2013;29:233-7.

28. Levi AA. Ovarian conservation during surgery with reference to bilateral dermoids and endometriosis. N Engl J Med. 1948;238:83-5.

29. Engel T, Greeley AV, Sweeney WJ 3^rd^. Recurrent dermoid cysts of the ovary. Report of 2 cases. Obstet Gynecol. 1965;26:757-9.

30. Lee DH. Coexistent mesenteric and ovarian mature cystic teratomas: a case report. Eur J Gynaecol Oncol. 2016;37:391-4.

31. Hegde P. Extragonadal omental teratoma: a case report. J Obstet Gynaecol Res. 2014;40:618-21.

32. Tokunaga M, Seta M, Yamada M, Nishio M, Yamamoto K, Koyasu Y. Coexistent dermoid cysts of the pouch of the Douglas and ovary resected by laparoscopy. Asian J Endosc Surg. 2012;5:31-3.

33. Yang WC, Wu MH, Chang FM, Kuo PL. Torsion of bilateral ovarian dermoid cysts with one parasitic teratoma at the omentum. Taiwanese J Obstet Gynecol. 2005;44:353-356.

34. Okino H, Koga Y, Tsuneyoshi M, Takeda S. Metachronous mature cystic teratomas in the left ovary and bilateral diaphragm: Report of a case. Surg Today. 2006;36:1012-4.

35. Kommoss F, Emond J, Hast J, Talerman A. Ruptured mature cystic teratoma of the ovary with recurrence in the liver and colon 17 years later. A case report. J Reprod Med. 1990;35:827-31.

36. Coskun A, Kiran G, Ozdemir O. CA 19-9 can be a useful tumor marker in ovarian dermoid cysts. Clin Exp Obstet Gynecol. 2008;35:137-9.

37. Vang R, Gown AM, Zhao C, Barry TS, Isacson C, Richardson MS, et al. Ovarian mucinous tumors associated with mature cystic teratomas: morphologic and immunohistochemical analysis identifies a subset of potential teratomatous origin that shares features of lower gastrointestinal tract mucinous tumors more commonly encountered as secondary tumors in the ovary. Am J Surg Pathol. 2007;31:854-69.

38. Dede M, Gungor S, Yenen MC, Alanbay I, Duru NK, Hasimi A. CA19-9 may have clinical significance in mature cystic teratomas of the ovary. Int J Gynecol Cancer. 2006;16:189-93.

39. Papadias K, Kairi-Vassilatou E, Kontogiani-Katsaros K, Argeitis J, Kondis-Pafitis A, Greatsas G. Teratomas of the ovary: a clinico-pathological evaluation of 87 patients from one institution during a 10-year period. Eur J Gynaecol Oncol. 2005;26:446-8.

40. Koçak M, Dilbaz B, Ozturk N, Dede S, Altay M, Dilbaz S, et al. Laparoscopic management of ovarian dermoid cysts: a review of 47 cases. Ann Saudi Med. 2004;24:357-60.

41. Sah SP, Uprety D, Rani S. Germ cell tumors of the ovary: a clinicopathologic study of 121 cases from Nepal. J Obstet Gynaecol Res. 2004;30:303-8.

42. Al-Fozan H, Glassman J, Caspi B, Appelman Z, Tulandi T. Lateral distribution of ovarian dermoid cyst. J Am Assoc Gynecol Laparosc. 2003;10:489-90.

43. Wu RT, Torng PL, Chang DY, Chen CK, Chen RJ, Lin MC, et al. Mature cystic teratoma of the ovary: a clinicopathologic study of 283 cases. Zhonghua Yi Xue Za Zhi (Taipei). 1996;58:269-74.

44. Ayhan A, Aksu T, Develioglu O, Tuncer ZS, Ayhan A. Complications and bilaterality of mature ovarian teratomas (clinicopathological evaluation of 286 cases). Aust N Z J Obstet Gynaecol. 1991;31:83-5.

45. Tarcoveanu E, Vasilescu A, Georgescu S, Danila N, Bradea C, Lupascu C, et al. Laparoscopic approach to ovarian dermoid cysts. Chirurgia (Bucur). 2012;107:461-8.

46. Saks M, Deckardt R. Laparoscopic Treatment of Benign Ovarian Dermoid Cysts. J Am Assoc Gynecol Laparosc. 1994;1:S31-2.

47. Malkasian GD Jr, Dockerty MB, Symmonds RE. Benign cystic teratomas. Obstet Gynecol. 1967;29:719-25.

48. Anteby EY, Ron M, Revel A, Shimonovitz S, Ariel I, Hurwitz A. Germ cell tumors of the ovary arising after dermoid cyst resection: a long-term follow-up study. Obstet Gynecol. 1994;83:605-8.

49. Pepe F, Panella M, Pepe G, Panella P, Pennisi F, Arikian S. Dermoid cysts of the ovary. Eur J Gynaecol Oncol. 1986;7:186-91.

50. Doss N Jr, Forney JP, Vellios F, Nalick RH. Covert bilaterality of mature ovarian teratomas. Obstet Gynecol. 1977;50:651-3.

51. Song YN, Zhu L, Lang JH. [Recurrent mature ovarian teratomas: retrospective analysis of 20 cases]. Zhonghua Yi Xue Za Zhi. 2007;87:1184-6.
